# Prognostic implications of autophagy-associated gene signatures in non-small cell lung cancer

**DOI:** 10.18632/aging.102544

**Published:** 2019-12-07

**Authors:** Yang Liu, Ligao Wu, Haijiao Ao, Meng Zhao, Xue Leng, Mingdong Liu, Jianqun Ma, Jinhong Zhu

**Affiliations:** 1 Department of Clinical Laboratory, Biobank, Harbin Medical University Cancer Hospital, Harbin 150040, Heilongjiang, China; 2 Department of Clinical Oncology, Harbin Medical University Cancer Hospital, Harbin 150040, Heilongjiang, China; 3 Department of Pathology, BengBu Medical College, BengBu 233000, Anhui, China; 4 Department of Thoracic Surgery, Harbin Medical University Cancer Hospital, Harbin 150040, Heilongjiang, China

**Keywords:** NSCLC, autophagy, prognosis, gene signature, nomogram

## Abstract

Autophagy, a highly conserved cellular proteolysis process, has been involved in non-small cell lung cancer (NSCLC). We tried to develop a prognostic prediction model for NSCLC patients based on the expression profiles of autophagy-associated genes. Univariate Cox regression analysis was used to determine autophagy-associated genes significantly correlated with overall survival (OS) of the TCGA lung cancer cohort. LASSO regression was performed to build multiple-gene prognostic signatures. We found that the 22-gene and 11-gene signatures could dichotomize patients with significantly different OS and independently predict the OS in TCGA lung adenocarcinoma (HR=2.801, 95% CI=2.252-3.486, *P*<0.001) and squamous cell carcinoma (HR=1.105, 95% CI=1.067-1.145, *P*<0.001), respectively. The prognostic performance of the 22-gene signature was validated in four GEO lung cancer cohorts. Moreover, GO, KEGG, and GSEA analyses unveiled several fundamental signaling pathways and cellular processes associated with the 22-gene signature in lung adenocarcinoma. We also constructed a clinical nomogram with a concordance index of 0.71 to predict the survival possibility of NSCLC patients by integrating clinical characteristics and the autophagy gene signature. The calibration curves substantiated fine concordance between nomogram prediction and actual observation. Overall, we constructed and verified a novel autophagy-associated gene signature that could improve the individualized outcome prediction in NSCLC.

## INTRODUCTION

Lung cancer is the most common cancer in the world, composed of 85% non-small cell lung cancer (NSCLC) and 15% small cell lung cancer (SCLC). Lung cancer alone led to about 1.6 million deaths in 2012, constituting 19% of all global cancer death [1, 2]. Several factors contribute to the unfavorable prognosis of lung cancer, including late diagnosis, inherent resistance to both chemotherapy and radiation therapy, acquired resistance to targeted therapy, and a high rate of relapse after the multimodal intervention [3]. To date, the prognostic prediction still mainly relies on histopathologic diagnosis and tumor staging system. However, the traditional approaches are not sufficient for precisely evaluating the outcomes of NSCLC patients. Therefore, it is imperative to develop robust and accurate prognostic biomarkers to help clinicians optimize therapeutic strategies. Over the past decades, considerable progress has been made in the understanding of tumor biology. One of the breakthrough findings is the involvement of macroautophagy (referred to thereafter as autophagy) in the development and therapeutic response of cancer [4–8].

Autophagy is a highly conserved catabolic cellular event degrading aggregated proteins and damaged organelles. The dynamic process includes induction, nucleation of the autophagosome, growth of the double-membrane, sealing and merging with the lysosome, and the disintegration of engulfed materials [6]. Typically, basal levels of autophagy ubiquitously exist in cells to break down and reuse non-functional cellular contents as an intracellular source of nutrients. In response to diverse stimuli and stresses, such as starvation, hypoxia, and drug, the magnitude of autophagy may increase dramatically to provide intracellular nutrients and remove harmful contents (e.g., damaged mitochondria) [5]. It suggests that autophagy is subjected to highly orchestrated regulation. Several signaling pathways known to regulate key cellular events are also implicated in autophagy, including PI3K/AKT/mTOR, p53/DRAM, JAK-STAT, RAS, and AMPK/CaMKK signaling pathways [6].

Autophagy is a double-edged sword in carcinogenesis, which either suppresses or promotes tumor development in a context-dependent manner, depending on tumor type, clinical stage, genetic background, and even therapeutic regimen. In general, autophagy is thought to prevent carcinogenesis by eradicating oncogenic protein substrates, misfolded proteins, and damaged organelles. However, in established cancer, active autophagic flux is often responsible for recycling intracellular macromolecules and organelles to fuel the extraordinary demands of tumor growth [7]. Increasing studies have demonstrated the implication of autophagy in NSCLC [6]. Autophagy is critical to the maintenance of glucose homeostasis and tumor growth in lung cancer [7]. Notably, in tumors with mutations in the RAS pathway genes, hyperactivity of autophagy is indispensable to meet extraordinarily high demands of tumor cell metabolism [9]. Consistently, lung tumors driven by the *Braf*^v600E^ mutation in mouse models were highly sensitive to autophagy inhibition [10]. Numerous studies have demonstrated that autophagy is involved in epidermal growth factor receptor tyrosine kinase inhibitor (EGFR-TKI) acquired resistance in NSCLC, partially due to the inhibition of PI3K/AKT/mTOR signaling pathway [11–14]. Moreover, high expression levels of autophagy-related gene 10 (ATG10) were associated with an unfavorable prognosis in lung cancer [15]. These findings substantiate the involvement of autophagy in lung cancer and suggest that autophagy-associated genes may hold great promise as prognostic markers in lung cancer.

However, autophagy is a complicated process involving hundreds of molecules. Therefore, compared to the single genes, a model integrating multiple autophagy-associated genes may increase prognosis predicting accuracy. In contrast to the traditional individual molecular prognostic predictors, genomic profiling based on “Omics” has provided an option to predict the prognosis of patients with a set of genes, known as “classifiers” or “signatures.” With this in mind, we used Cox proportional hazard regression analysis to screen prognosis-related genes out of 148 genes autophagy-associated in The Cancer Genome of Atlas (TCGA) lung cancer cohort. And then, the resulting genes were applied to the least absolute shrinkage and selection operator (LASSO) to establish an optimal risk model, followed by validation in several independent GEO lung cancer populations. Patients were divided into high and low risk groups by the median risk score. Survival analysis was carried out to assess the prognostic values of the risk score. The differences in the critical signaling pathways between high and low risk groups were explored using Gene Ontology (GO), Kyoto Encyclopedia of Genes and Genomes (KEGG), and Gene Set Enrichment Analysis (GSEA). Finally, a nomogram was built to predict the individuals’ survival probability by integrating clinical characteristics and the prognostic gene signature.

## RESULTS

### Characteristics of patients

TCGA lung cancer cohorts consisted of a total of 490 lung adenocarcinoma (LUAD) and 488 squamous cell lung cancer (LUSC) patients. The Demographic and clinical features of patients were listed in Supplementary Table 1. Kaplan-Meier survival curves were plotted for LUAD and LUAC cohorts regarding tumor (T), lymph (N), metastasis (M), and TNM stage (Supplementary Figure 1).

### Construction of prognostic signature for TCGA lung cancer cohorts

**Figure 1:**
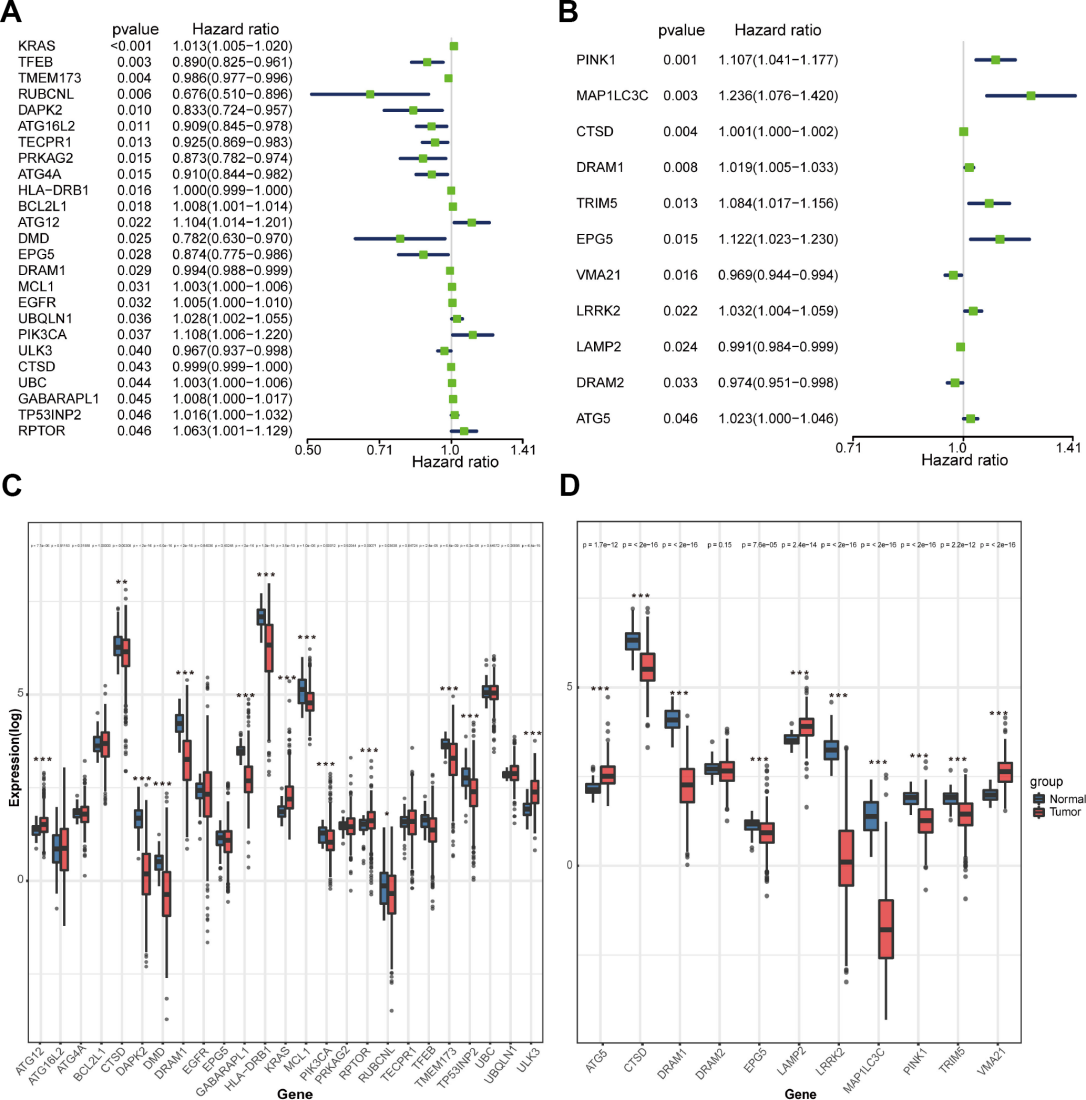
**Selection of autophagy genes associated with the survival of lung cancer by univariate Cox regression analysis.** (**A**) Forest plot of autophagy genes associated with TCGA-LUAD survival. (**B**) Forest plot of autophagy genes associated with TCGA-LUSC survival. (**C**) Differential expression of the 25 selected genes between normal and LUAD tissues. (**D**) Differential expression of the 11 selected genes between normal and LUSC tissues.

**Figure 2:**
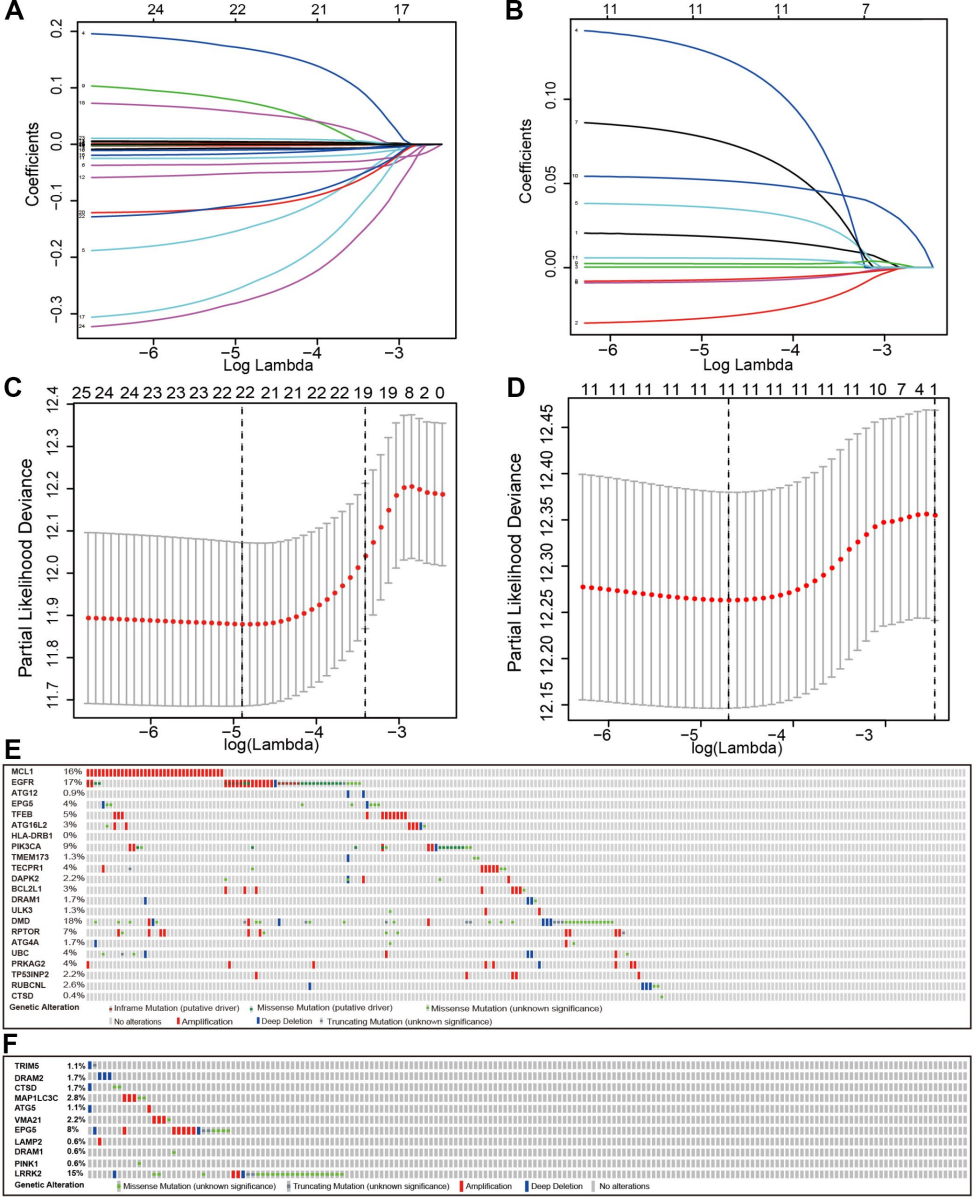
**Establishment of prognostic gene signature by LASSO regression analysis.** LASSO coefficient profiles of the 25 genes in TCGA-LUAD (**A**) and 11 genes in TCGA-LUSC (**B**). A coefficient profile plot was generated against the log (lambda) sequence. Selection of the optimal parameter (lambda) in the LASSO model for TCGA-LUAD (**C**) and TCGA-LUSC (**D**). (**E**) Genetic alteration of the 22 genes in the TCGA-LUAD cohort (TCGA, Provisional). (**F**) Genetic alteration of the 11 genes in the TCGA-LUSC cohort (TCGA, Provisional).

We searched genes associated with autophagy in the GeneCards database. A total of 149 autophagy-associated genes with relevance score >7 were chosen to generate prognostic gene signatures. *XBP1* was removed due to a lack of expression in TCGA lung cohorts. Gene expression profiles of 148 genes in normal and tumor tissues were separately displayed for TCGA-LUAD and TCGA-LUSC cohorts in the heatmaps (Supplementary Figure 2). All these genes were subjected to univariate Cox regression analysis. A total of 25 and 11 genes were significantly associated with the OS of TCGA-LUAD (Figure 1A and 1C) and TCGA-LUSC (Figure 1B and 1D), respectively. These significant genes entered into LASSO COX regression analysis, and the regression coefficient was computed. Coefficient of each gene in LUAD was illustrated in Figure 2A. While 22 genes were included, the model achieved the best performance (Figure 2C). Similar analyses were performed for the TCGA-LUSC cohort, ending up with 11 genes significantly associated with survival (Figure 2B, 2D, and 2F). The functions, coefficients, and relevance scores of these genes were shown in Table 1, which included signal transduction molecules, components of autophagosome and lysosome, as well as enzymes facilitating the formation of autophagosomes.

**Table 1:** Functions of genes in the prognostic gene signatures.

**Type**	**No**	**Gene symbol**	**Full name**	**Function**	**Risk coefficient**	**Relevance Score**
LUAD	1	RUBCNL	Rubicon Like Autophagy Enhancer	Promotes autophagosome maturation	-0.28125	14.2
	2	DMD	Dystrophin	*Autophagy*-related proteins	-0.25704	12.01
	3	DAPK2	Death Associated Protein Kinase 2	Trigger cell survival, apoptosis, and *autophagy*	-0.05171	7.65
	4	PRKAG2	Protein Kinase AMP-Activated Non-Catalytic Subunit Gamma 2	*Autophagy*-Related Proteins	-0.10836	7.13
	5	EPG5	Ectopic P-Granules Autophagy Protein 5 Homolog	Clearance of autophagosomal cargo	-0.15368	17.36
	6	TFEB	Transcription Factor EB	Specifically recognizes lysosomal genes	-0.03524	12.12
	7	ATG16L2	Autophagy Related 16 Like 2	*Autophagy*-Related Proteins	-0.00683	21.99
	8	ATG4A	Autophagy Related 4A Cysteine Peptidase	*Cysteine protease required for autophagy*	-0.11242	28.20
	9	TECPR1	Tectonin Beta-Propeller Repeat Containing 1	Tethering factor involved in autophagy	-0.02462	9.24
	10	ULK3	Unc-51 Like Kinase 3	Induce *autophagy*	-0.01695	8.92
	11	TMEM173	Transmembrane Protein 173	*Play role in* immune signaling and autophagy	-0.00993	7.11
	12	DRAM1	DNA Damage Regulated Autophagy Modulator 1	Lysosomal modulator of *autophagy*	-0.0024	22.24
	13	CTSD	Cathepsin D	*Autophagy*-related protein	-0.00014	8.25
	14	HLA-DRB1	Major Histocompatibility Complex, Class II, DR Beta 1	*Autophagy*-related protein	-4.95E-05	8.69
	15	UBC	Ubiquitin C	A polyubiquitin precursor	-0.00031	7.13
	16	MCL1	MCL1, BCL2 Family Apoptosis Regulator	Anti-apoptotic protein	0.004578	8.11
	17	EGFR	Epidermal Growth Factor Receptor	Regulation of autophagy	0.001047	7.09
	18	BCL2L1	BCL2 Like 1	Anti- or pro-apoptotic regulators	0.004578	7.99
	19	TP53INP2	Tumor Protein P53 Inducible Nuclear Protein 1	Dual regulator of transcription and autophagy.	0.009989	13.27
	20	RPTOR	Regulatory Associated Protein Of MTOR Complex 1	*Autophagy*-Related Protein	0.057963	8.91
	21	ATG12	Autophagy- related 12	Ubiquitin-like protein involved in *autophagy* vesicles formation.	0.171853	34.22
	22	PIK3CA	Phosphatidylinositol-4,5-Bisphosphate 3-Kinase Catalytic Subunit Alpha	*Autophagy*-related proteins	0.077961	8.59
LUSC	1	DRAM2	DNA Damage Regulated Autophagy Modulator 2	Plays a role in the initiation of *autophagy*	-0.03131	19.68
	2	VMA21	Vacuolar ATPase Assembly Factor VMA21	*Autophagy*-related proteins	-0.0087	47.93
	3	LAMP2	Lysosomal Associated Membrane Protein 2	Plays an important role in chaperone-mediated *autophagy*	-0.00761	39.19
	4	CTSD	Cathepsin D	*Autophagy*-related protein	0.000315	8.25
	5	DRAM1	DNA Damage Regulated Autophagy Modulator 1	Lysosomal modulator of *autophagy*	0.002342	22.24
	6	LRRK2	Leucine Rich Repeat Kinase 2	Positively regulates *autophagy*	0.005741	9.36
	7	TRIM5	Tripartite Motif Containing 5	Activation of *autophagy* regulator BECN1	0.019368	7.57
	8	ATG5	Autophagy Related 5	*Autophagy*-related protein	0.036959	40.78
	9	PINK1	PTEN Induced Kinase 1	*Autophagy* of mitochondrion	0.053167	9.14
	10	EPG5	Ectopic P-Granules Autophagy Protein 5 Homolog	Clearance of autophagosomal cargo	0.081189	17.36
	11	MAP1LC3C	Microtubule Associated Protein 1 Light Chain 3 Gamma	Senescence and *Autophagy* in Cancer	0.134076	17.15

We examined the genetic alteration of these risk-associated genes in lung cancer to understand their contributions to lung carcinogenesis (http://www.cbioportal.org). Datasets of Provisional and PanCancer Atlas for LUAD or LUSC were applied (Lung Adenocarcinoma: 586 samples in Provisional vs. 566 samples in PanCancer Atlas; Lung Squamous Cell Carcinoma: 511 samples in Provisional vs. 487 samples in PanCancer Atlas). Only patients/samples harboring both mutations and CAN data were included. In terms of LUAD, genes of interest are altered in 289 (57%) of 507 queried patients/samples (PanCancer Atlas) (supplementary Figure 3A), compared with that altered queried genes were detected in 151 (66%) of 230 patients/samples (Provisional) (Figure 2E). In terms of LUSC, queried genes are changed in 144 (31%) of 469 queried patients/samples (PanCancer Atlas) (supplementary Figure 3B), compared with 52 (29%) of 178 TCGA-LUSC patients/samples (Provisional) (Figure 2F). The frequent genetic alterations suggested the crucial roles of these genes in the development of lung cancer.

**Figure 3:**
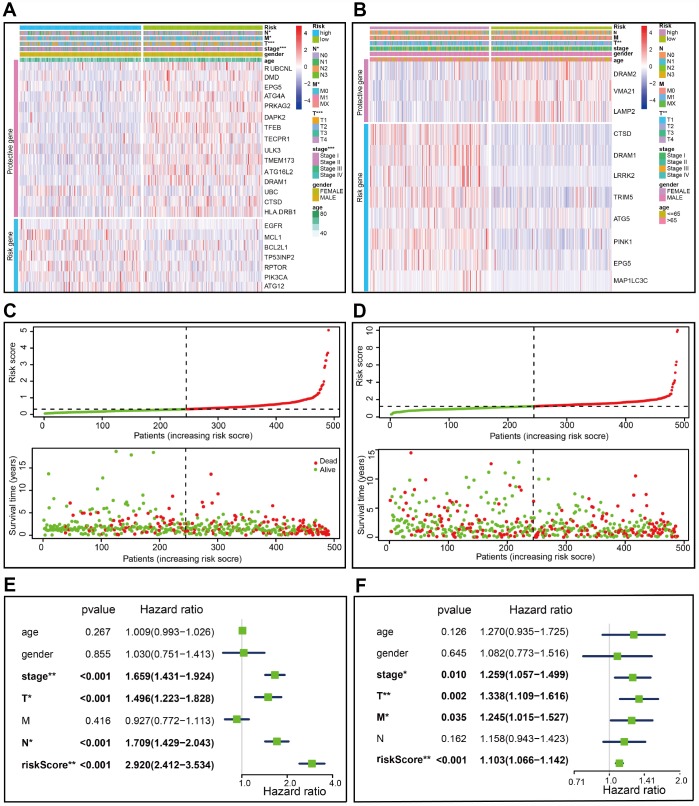
**Characteristics of the prognostic gene signature.** (**A**–**B**) Heatmap of the autophagy-associated gene expression profiles in prognostic signature for TCGA-LUAD (**A**) and TCGA-LUSC (**B**). (C–D) The distribution of risk score and patient’s survival time, as well as status for TCGA-LUAD (**C**) and TCGA-LUSC (**D**). (**C**) The black dotted line is the optimum cutoff dividing patients into low risk and high risk groups. (**E**–**F**) Univariate Cox regression analysis. Forest plot of the association between risk factors and survival of TCGA-LUAD (**E**) or TCGA- LUSC (**F**).

A risk score was computed for each patient formulated on the mRNA expression level and risk coefficient of each gene; that is, a linear combination of the mRNA level of each autophagy-associated gene weighted by its multivariable LASSO regression coefficient. The risk score was applied to predict prognosis, with the median risk score as a cutoff value to separate patients into high and low risk groups. A heatmap was plotted to show the gene expression profiles in high and low risk LUAD groups (Figure 3A). Genes (*EGFR*, *MCL1*, *BCL2L1*, *TP53INP2*, *RPTOR*, *PIK3CA*, and *ATG12*) with HR>1 were considered as risk genes, while those (*RUBCNL*, *DMD*, *EPG5*, *ATG4A*, *PRKAG2*, *DAPK2*, *TFEB*, *TECPR1*, *ULK3*, *TMEM173*, *ATG16L2*, *DRAM1*, *UBC*, *HLA-DRB1*, and *CTSD*) with HR<1 as protective genes (Figure 3A).

**Figure 4:**
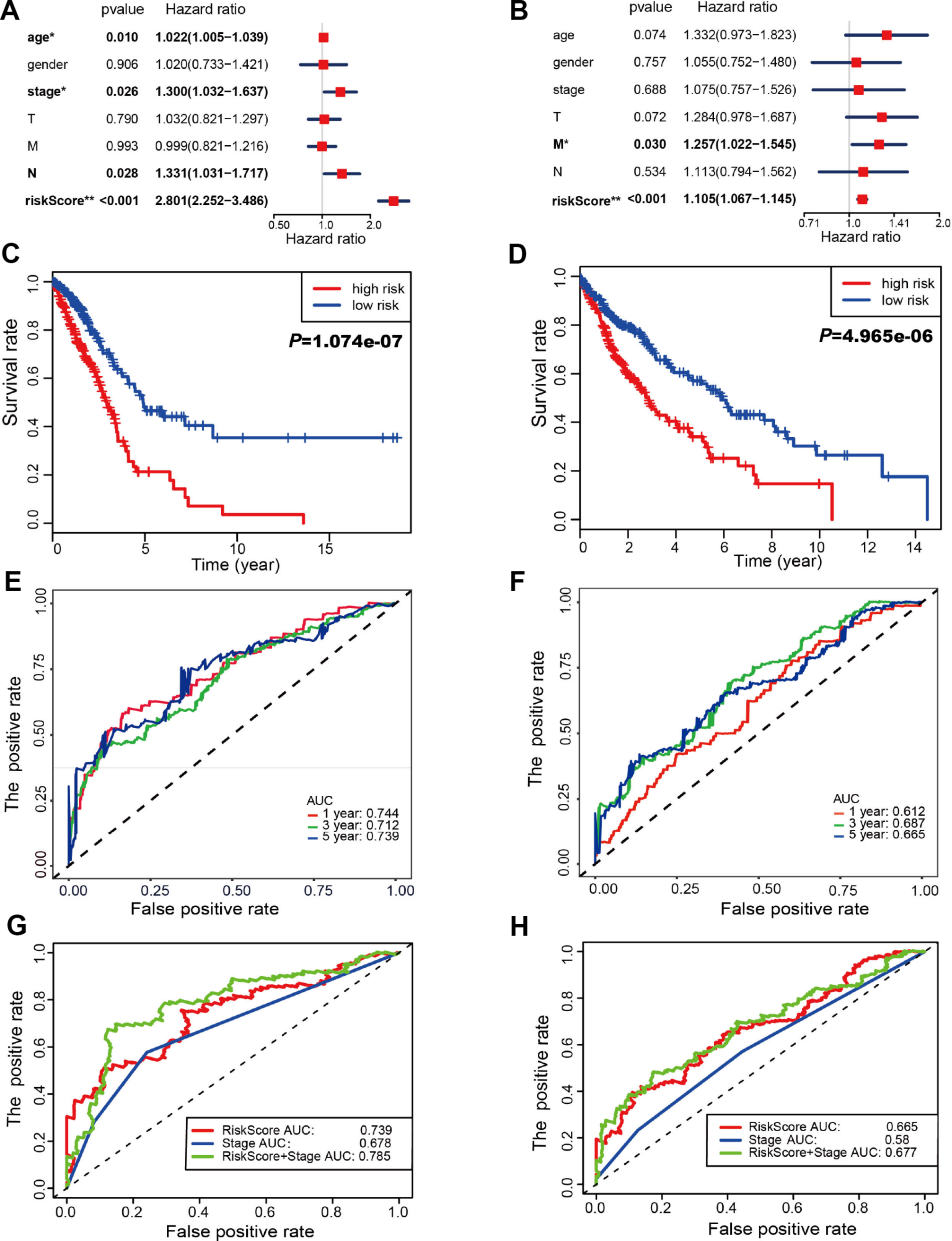
**Autophagy-associated gene signature was significantly related to survival in lung cancer.** (**A**–**B**) Multivariate Cox regression analysis. The autophagy-associated gene signature was an independent predictor of prognosis in TCGA-LUAD (**A**) and TCGA- LUSC (**B**). (**C**–**D**) Kaplan-Meier analysis of TCGA lung cancer patients stratified by the median risk score. (**C**) The high risk scores were related to poor overall survival in TCGA-LUAD. (**D**) The high risk scores were correlated with poor overall survival in TCGA-LUSC. (**E**–**F**) Receiver operating characteristic (ROC) analysis of the sensitivity and specificity of the OS for the 22-gene risk score in TCGA-LUAD (**E**) and 11-gene risk score in TCGA-LUSC (**F**). The combination of stage and risk score could better predict prognosis in TCGA-LUAD (**G**) and TCGA-LUSC (**H**) than either one alone.

Risk scores were significantly associated with T, N, M, and clinical stage in TCGA-LUAD cohorts (Figure 3A). As illustrated, patients in the high risk group were more likely to express risk genes. In contrast, patients in the low risk group had a tendency to express protective genes (Figure 3A). The distributions of risk score of LUAD patients and the relationships between risk score and survival time were visualized in Figure 3C. Similar analyses were performed for TCGA-LUSC cohorts (Figure 3B and 3D). Following that, we evaluated the prognostic value of the risk score. Regarding TCGA-LUAD, risk scores were significantly associated with overall survival (OS) (HR=2.920, 95% CI=2.412-3.534, *P*<0.001) in the univariate analysis (Figure 3E). Multivariate analysis revealed that the risk score was an independent prognostic predictor (HR=2.801, 95% CI=2.252-3.486, *P*<0.001) (Figure 4A). Kaplan-Meier cumulative curves indicated that patients with low risk scores survived significantly longer than those with high risk scores (Figure 4C). The risk score was also associated with TCGA-LUSC cohort, as evidenced by univariate (HR=1.103, 95% CI=1.066-1.142, *P*<0.001) (Figure 3F) and multivariate (HR=1.105, 95% CI=1.067-1,145, *P*<0.001) Cox regression analyses (Figure 4B), as well as Kaplan-Meier survival curve (Figure 4D). The highest area under the curve (AUC) values of the risk score was 0.744 and 0.684 for the TCGA-LUAD and -LUSC cohorts, respectively (Figure 4E and 4F). Twenty-two autophagy-associated genes in LUAD were used for further analysis since they surpassed those genes in LUSC in predicting prognosis. The combination of stage and risk score could improve prognostic accuracy in TCGA-LUAD (Figure 4G) and TCGA-LUSC (Figure 4H) when compared to the stage or risk score alone.

### Identification of involved signaling pathways

**Figure 5:**
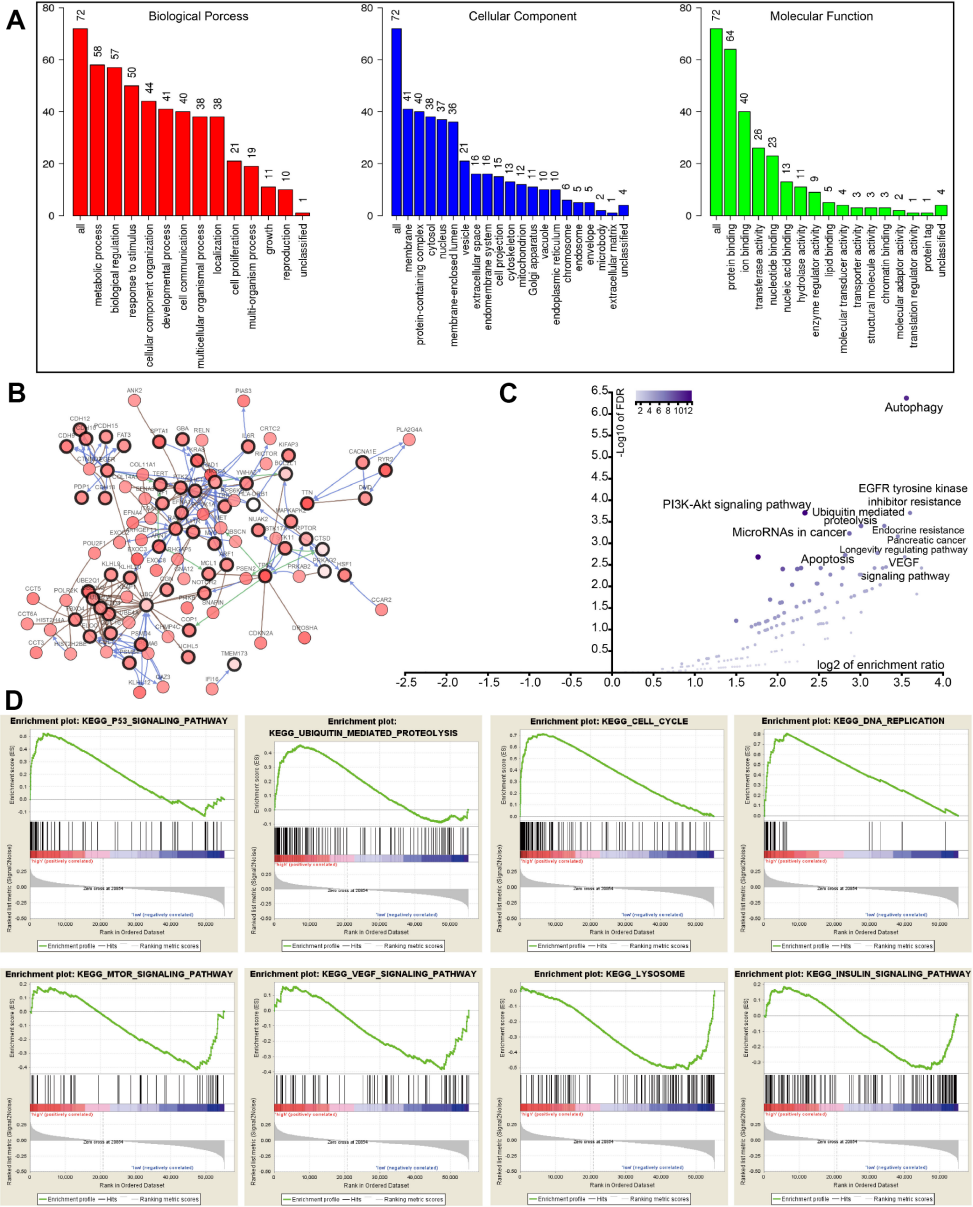
**GO, KEGG, and GSEA analysis.** (**A**) GO analysis of 22 autophagy-associated genes and 50 altered neighbor genes. (**B**) Proteins interacted with the 22 autophagy-associated genes (black circle) in TCGA-LUAD. (**C**) Volcano of autophagy genes-associated pathways. (**D**) GSEA analysis of the differentially expressed genes between high and low risk groups.

To interrogate potential signaling pathways related to the 22 autophagy-associated genes in lung cancer, we used them as baits to hook 50 most frequently changed neighbor genes in the TCGA-LUAD cohort (http://www.cbioportal.org). GO analysis indicated that these genes could be categorized into several essential biological processes, including biological regulation, response to the stimulus, developmental process, cell proliferation, cell proliferation, and growth (Figure 5A). These genes were linked and formed a tight protein-protein interaction network as indicated in Figure 5B (https://string-db.org/). KEGG analysis showed that the 72 genes were mainly associated with autophagy, apoptosis, EGFR tyrosine kinase inhibitor (TKI) resistance, ubiquitin-mediated proteolysis, PI3K-Akt signaling pathway, and VEGF signaling pathway (Figure 5C). The log2 of enrichment ratio and -log10 of FDR were visualized in the volcano plot (Figure 5C). Fold changes of mRNA expression levels of all protein-coding genes between high and low risk LUAD groups were calculated and pre-ranked from high to low. GSEA analysis unveiled that altered genes were significantly enriched in several common pathways (Figure 5D). We found that the high risk group was significantly associated with the cell cycle (NES=2.80, P<0.0001), p53 signaling pathway (NES=1.937, *P*=0.0039), DNA replication (NES=1.90, *P*=0.0059), and ubiquitin-mediated proteolysis (NES=1.703, *P*=0.017). Meanwhile, the low risk group was negatively associated with mTOR (NES=-1.526, *P*=0.045), VEGF (NES=-1.529, P=0.033), and insulin (NES=-1.424, *P*=0.044) signaling pathways, as well as lysosome (NES=-1.747, *P*=0.024).

### Validation of the prognostic gene signature in the independent lung cancer cohorts

**Figure 6:**
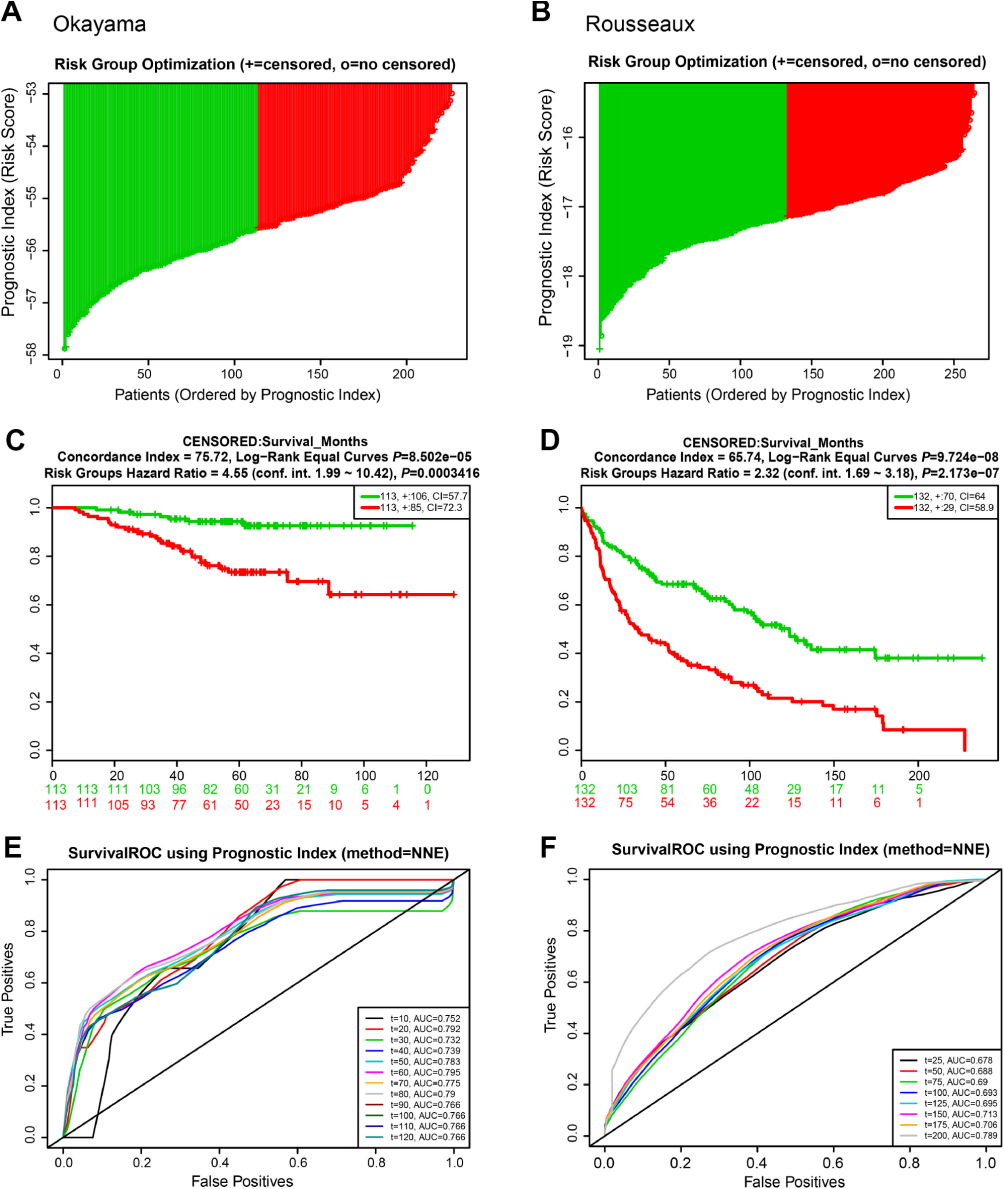
**Risk scores of 22-autophagy gene signature were significantly associated with survival in the Okayama and Rousseaux cohorts.** The distribution of risk score (**A**), Kaplan-Meier survival curve (**C**), and ROC curve (**E**) for the Okayama cohort. The distribution of risk score (**B**), Kaplan-Meier survival curve (**D**), and ROC curve (**F**) for the Rousseaux cohort.

**Figure 7:**
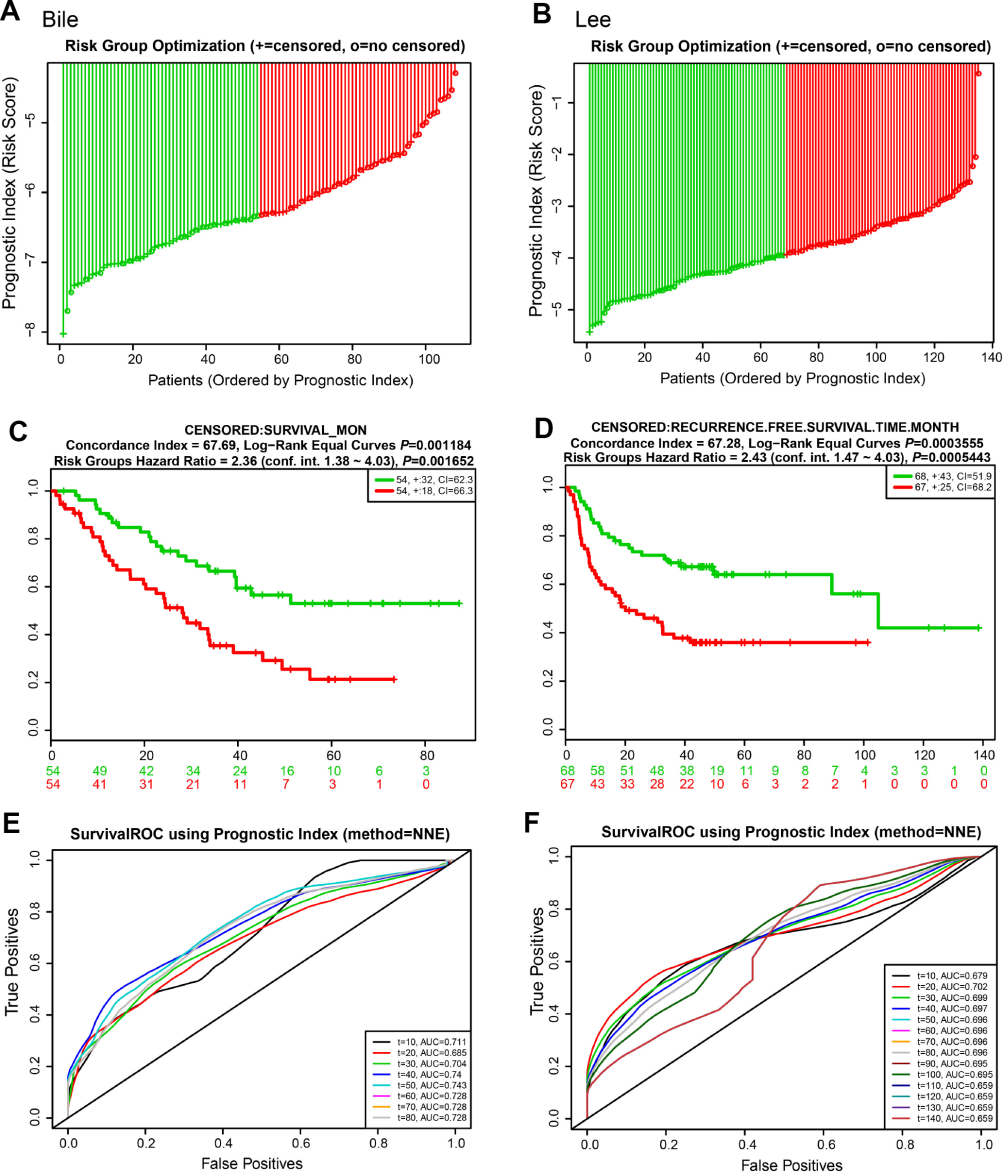
**Risk scores of 22-autophagy gene signature were significantly associated with survival in the Bile and Lee cohorts.** The distribution of risk score (**A**), Kaplan-Meier survival curve (**C**), and ROC curve (**E**) for the Bile cohort. The distribution of risk score (**B**), Kaplan-Meier survival curve (**D**), and ROC curve (**F**) for the Lee cohort.

We next evaluated the predictive power of the prognostic gene signature in the different lung cancer cohorts from the GEO database [16]. In each cohort, patients were separated into low and high risk groups based on the calculated risk score, and the OS of the two groups was compared. Okayama cohort consisted of 226 patients with primary stage I-II lung adenocarcinoma (GSE31210) [17] (Figure 6). Risk scores of patients spanned from -58 to -53 (Figure 6A). Patients at low risk survived significantly longer than those in high risk (HR=4.55, 95% CI=1.99-10.42, *P*=0.0003416, and maximum AUC=0.795) (Figure 6C and 6E). Rousseaux cohort included 293 patients with stage I-IV lung cancer (GSE30219), consisting of 71 adenocarcinomas, 61 squamous cell tumors, 56 large cell neuroendocrine tumors, 39, Basaloid tumors, 24 carcinoid tumors, 21 small cell carcinoma, and 7 other histology [18]. Risk scores ranged from -19 to -15 (Figure 6B). The risk scores performed well, even in this mixed lung cancer cohort (HR=2.32, 95% CI=1.69-3.18, *P*=2.173e-07, maximum AUC=0.789) (Figure 6D and 6F). In the Bild cohort of 109 lung cancer patients (GSE3141) [19], patients with smaller risk scores outperformed those with high risk in survival (HR=2.36, 95% CI=1.38-4.03, *P*=0.001652, maximum AUC=0.743) (Figure 7A, 7C, and 7E). Lastly, Lee’s study (GSE8894) was performed in 138 patients with stage IA-IIIB postoperative NSCLC (adenocarcinoma and squamous cancer cell lung cancer) [20]. Patients in the low risk group exhibited longer OS than the high risk group (HR=2.43, 95% CI=1.47-4.03, *P*=0.0005443, maximum AUC=0.702) (Figure 7B, 7D, and 7F). Overall, these results confirmed that this 22-autophagy gene signature was also predictive of survival in the independent validation lung cohorts.

### A personalized prognostic prediction model

**Figure 8:**
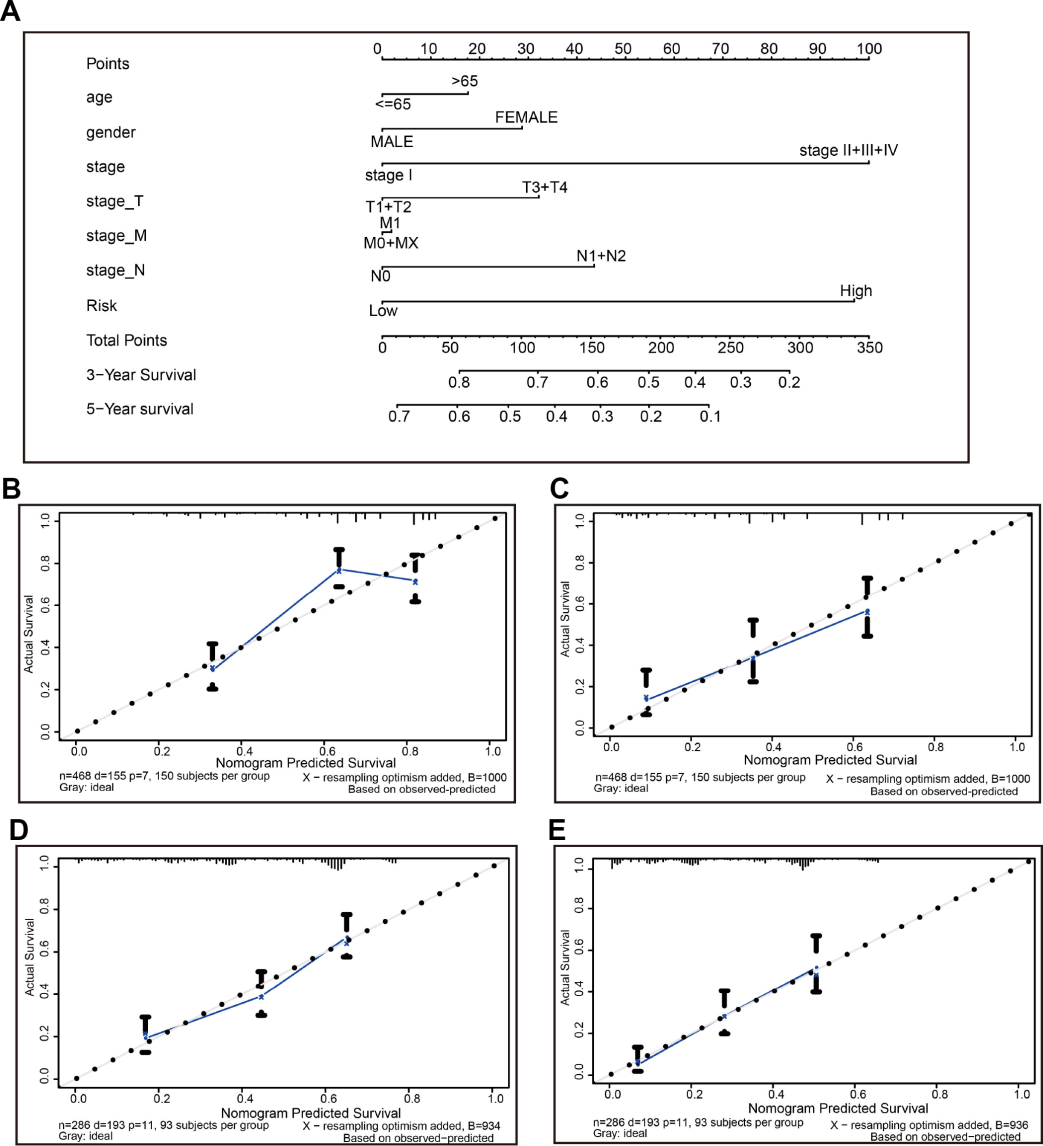
**The nomogram to anticipate prognostic probabilities in TCGA-LUAD.** (**A**) The nomogram for predicting OS developed TCGA-LUAD cohort (training set). (**B**–**C**) The calibration plots for predicting 3-year (**B**) and 5-year survival (**D**) in the training set. The calibration plots of 3-year (**D**) and 5-year survival (**E**) in the GSE30219 lung cancer cohort (testing set). The x-axis and y-axis represented nomogram-predicted and actual survival, respectively. The solid line indicated the predicted nomogram and the vertical bars represent a 95% confidence interval.

A nomogram is a robust tool that has been applied to quantitatively determine individuals’ risk in the clinical setting by integrating multiple risk factors [21–25]. We generated a nomogram to predict the probability of 3- and 5-year OS, by incorporating the 22-autophagy gene signature, age, gender, T, N, M, and TNM stage. As shown in Figure 8A, each factor was assigned points in proportion to its risk contribution to survival. Calibration curves indicated that actual and predicted survival matched very well (Figure 8B and 8C), especially for 5-year survival. For instance, a 70-year old (17.5 points) female patient (30 points) would obtain a total of 219 points, if she had stage I (0 points), stage_T3/4 (32 points), stage_M0 (0 points), and stage N1/2 disease (42 points), as well as high risk score (97.5 points). Her 3-year and 5-year survival was about 42% and 12%, respectively. The nomogram was validated in the GSE30219 lung cancer cohort, and 3- and 5- year calibration curves were presented in Figure 8D and Figure 8E, respectively.

## DISCUSSION

Autophagy is a multiple-step process tightly regulated by several fundamental signaling pathways. It requires the sequential formation of two molecular complexes: the ULK1 complex [UNC-51-like kinase 1 (ULK1), ULK2, FAK family a kinase-interacting protein of 200 kDa (FIP200), ATG13L, and ATG101)] and the class III PI3K complex [vacuolar protein sorting 15 (VPS15), VPS34, Beclin-1, ATG14 or UV radiation resistant-associated gene protein (UVRAG), and activating molecule in BECN1-regulated autophagy protein 1 (AMBRA1)]. Moreover, two ubiquitin-like conjugation systems are responsible for the expansion and elongation of the autophagosome membrane: microtubule-associated protein type 1 light chain 3 (MAP1LC3) -lipid phosphatidylethanolamine (PE) conjugation system and ATG5-ATG12-ATG16L.

Several studies have suggested the potential association between autophagy proteins and NSCLC survival. High expression levels of ATG10 were associated with an unfavorable prognosis in lung cancer [15]. In a Czech Republic population with IB-III NSCLC, patients with more LC3A-positive stone-like structures were significantly more likely to have the poor OS and disease-free survival (DFS) [26]. To date, hundreds of proteins are considered to be involved in the autophagy process. Given the importance of autophagy in lung cancer, it is reasonable to speculate that autophagy-associated genes hold great promises in prognostic prediction and that multiple-gene signature derived from reliable algorithms would be superior to single molecules in predicting OS of NSCLC.

In this study, we profiled the mRNA expression of 148 autophagy-associated genes in the TCGA lung cancer cohort. Among them, 25 genes were associated with the survival of TCGA-LUAD, while 11 genes with the survival of TCGA-LUSC. We used LASSO regression to develop a 22-gene and an 11-gene prognostic signature for TCGA-LUAD and -LUSC cohort, respectively. The risk score was calculated for each patient by integrating mRNA expression levels and risk coefficients for selected genes. The risk scores significantly stratified patient outcomes in both TCGA-LUAD and -LUSC cohorts. More importantly, the prognostic power of the 22-gene signature was validated in several independent GEO lung cohorts comprising of both LUAD and LUSC, as well as early-stage lung cancer populations. As early as 2007, in an attempt to develop gene signatures correlated with NSCLC clinical outcomes, Chen et al. developed a five-gene prognostic signature for NSCLC [27]. Since then, gene signature, also known as classifier, has been frequently used to predict prognosis in a variety of tumors [28–31], which even outperformed the TNM staging method in some cases [29]. Prognostic gene signatures based on autophagy-associated genes have been reported in colon cancer, colorectal cancer, serous ovarian cancer, and hepatocellular carcinoma [30, 32–34]. For instance, following our results, Mo and colleagues recently reported a nine-autophagy-related signature (*CAPN2*, *ATG16L2*, *TP63*, *SIRT1*, *RPS6KB1*, *PEX3*, *ATG5*, *UVRAG*, *NAF1*) based on relapse-free survival in patients with resected stage I-III colon cancer [30]. This gene signature successfully distinguished patients at a significantly increased risk of early relapse from those at low risk [30]. When we compared our gene signature with Mo’s, few genes were in common between the two datasets, suggesting that the contributing autophagy-associated genes were tumor type-specific.

Bioinformatic approach uncovered that the 22-gene signature mainly associated with the PI3K-AKT signaling pathway, the VEGF signaling pathway, EGFR tyrosine kinase inhibitor (EGFR-TKI) resistance, apoptosis, and ubiquitin-mediated proteolysis in LUAD. Interestingly, tons of studies have found that autophagy plays a part in the EGFR-TKI resistance of tumor cells [11–14, 35]. EGFR-TKI induces autophagy through the suppression of the PI3K/AKT/mTOR signaling pathway, which in turn saves tumor cells from the harm of EGFR-TKIs [14]. Inversely, inhibition of autophagy overcame the resistance of tumor cells to EGFR-TKIs [14]. There is also numerous evidence supporting the interplay between autophagy and apoptosis [36–38]. Autophagy may either promote or hinder apoptosis.

On the one hand, autophagy facilitates cells to survive the stressful condition by providing essential nutrients and eliminating damaged organelles; on the other hand, excessive autophagy may lead cells to death, namely autophagic cell death (ACD) [37, 39]. Some molecules regulating both apoptosis and autophagy may underlie the crosstalk between these two processes. For instance, Beclin-1 is known to interact with anti-apoptotic Bcl-2 protein to suppress both autophagy and apoptosis at basal levels. However, upon nutrient starvation, Beclin-1 was released from the binding to Bcl-2, and in turn, formed an autophagic complex with VPS34 and other molecules to promote autophagy progression; meanwhile, the resulting phosphorylated Bcl-2 prevented apoptosis by binding to Bax [40].

Interestingly, apoptotic protein caspases were found to be involved in the regulation of autophagy [41]. Activated caspases could cleave autophagy-associated proteins (e.g., Beclin-1, ATG5, and p62), thereby inhibiting autophagy [42]. Several lines of evidence suggested that the understanding of the crosstalk between autophagy and apoptosis in NSCLC may facilitate clinicians to make better therapeutic strategies. Salinomycin was reported to induce apoptosis of NSCLC cells through inhibiting the AKT1-mTOR signaling pathway, accompanied by the activation of autophagy; blockage of autophagy augmented salinomycin-mediated apoptosis, suggesting that the autophagic response plays cytoprotective roles [43]. It was also the case with anti-EGFR treatments, as mentioned above. Autophagy inhibitors have been demonstrated to increase the sensitivity of NSCLC cells to EGFR-TKIs [35]. Moreover, the molecular interplay among apoptosis, autophagy, and proteasomal protein degradation pathway are also well documented [38, 44]. Consistently, GSEA analysis also indicated the involvement of mTOR, VEGF, and ubiquitin-mediated proteolysis, and further discerned the participation of cell cycle, DNA replication, the p53 signaling pathway, the insulin signaling pathway, and lysosome in the development of LUAD.

Lastly, we developed a nomogram to predict individuals’ clinical outcomes. A nomogram is a steady and credible tool to quantitatively measure risk on an individual basis by combining and delineated risk factors, which has been used for oncology prognoses, including NSCLC [21–25]. A nomogram generates a statistical predictive model presented in a graph, conferring points to each factor such as age, gender, and TNM stage in the clinical setting. By summarizing all the points, this model yields a numerical possibility for an individual regarding a clinical outcome, such as overall survival (OS), relapse, and medication nonadherence. Apart from traditional clinicopathological features (e.g., TNM stage, tumor size, and histological subtype), risk score derived from the gene signature could also be incorporated into a predictive nomogram model to better predict clinical outcomes [28–30, 45, 46]. Mo et al. reported a nomogram to predict 3-, 5-, and 7- year relapse-free survival in colorectal cancer with the inclusion of a prognostic score calculated from autophagy-gene signature [30]. The combination of the autophagy-gene signature and prognostic factors achieved better prognostic performance than each one alone [30]. Xiong and colleagues demonstrated the improved prognosis prediction of colorectal cancer with the introduction of a lncRNA-microRNA-mRNA signature [29]. The multi-RNA-based signature exhibited high prognostic accuracy than the TNM stage. Moreover, as demonstrated by the calibration curve, the nomogram adopting both the RNA signature and conventional prognostic factors could accurately predict 3-year and 5-year survival probabilities [29]. Despite the popularity of the integration of gene signature and traditional prognostic factors in predicting prognosis of colon cancer, the application of this strategy in the NSCLC is striking limited [28]. Wang and colleagues demonstrated a novel lymph node metastasis-related gene signature to forecast OS for LUAD patients with lymph node metastasis and the fine use of a nomogram combining gene signature with clinicopathological features [28]. Consistently, in the current study, we indicated that a nomogram, including a 22-autophagy gene signature, could well predict 3- and 5- year survival possibilities of LUAD patients.

In conclusion, we identified a prognostic 22-autophagy gene signature based on TCGA and GEO lung cancer cohorts. This gene signature was an independent predictor of prognosis. A nomogram based on the gene signature and clinicopathological feature could accurately predict a 3- and 5-year survival probability for individual lung cancer patients. Our finding suggests that the 22-autophagy gene signature may help facilitate personalized medicine in the clinical setting.

## MATERIALS AND METHODS

### Selection of autophagy-associated genes

Autophagy-associated genes were retrieved by searching the GeneCards website (https://www.genecards.org/) with the term “autophagy.” A relevance score was used to indicate the strength of the correlation between genes and autophagy activity, ranging from 0 to 100. Lager scores represent the stronger associations. Genes with association score >7 were taken into as autophagy-associated genes.

### Acquisition of lung cancer datasets

Both raw RNA-sequencing (RNA-seq) datasets and clinical characteristics of TCGA lung cohorts were downloaded from the TCGA website (https://portal.gdc.cancer.gov/). R version 3.6.0 software was used to normalize and process the data. GSE31210, GSE30219, GSE3141, and GSE3141 datasets were obtained from the Gene Expression Omnibus (GEO, https://www.ncbi.nlm.nih.gov/geo/) for the validation studies.

### Identification and validation of the prognostic gene signature

Univariate Cox proportional hazard regression analysis was conducted to screen the autophagy genes significantly associated with overall survival (OS) of TCGA-lung cancer cohorts. Identified OS-associated genes were used to develop prognostic multiple-gene signatures. Autophagy-associated gene signatures (minimal length best performing multivariate models) were built for OS. The least absolute shrinkage and selection operator (LASSO) Cox regression method was adopted to construct multivariable models with autophagy-related genes using the “glmnet” package for R [47, 48]. LASSO regression achieves dimension reduction of high-dimensional data by restricting the sum of the absolute value of coefficients to be smaller than a predetermined value. As a result, variables with a relatively small contribution would be conferred a coefficient of zero. The best model was determined by maximizing model performance and minimizing the number of features (i.e., genes). Only genes with nonzero coefficients in the LASSO regression model were chosen to further calculate the risk score [49]. We computed risk score for each patient using the following formula: risk scores $=\overset{n}{\underset{j=1}{\sum }}Coefj*Xj$, with Coef_j_ indicating the coefficient and x_j_ representing the relative expression levels of each autophagy-related gene standardized by z-score. The median risk score was chosen as a cutoff value to separately dichotomize TCGA-LUAD and -LUSC cohorts. The prognostic gene signature was verified in the four independent lung cancer cohorts (GSE31210, GSE30219, GSE3141, and GSE8894) [16]. The same formula was used to calculate risk scores in GEO datasets, as in the TCGA datasets.

### Pathway analysis

The functional annotation of Gene Ontology (GO), including biological process, cellular component, and molecular function, was performed using the open access WebGestalt tool (http://www.webgestalt.org) [50, 51]. The same tool was also used to implement the KEGG pathway enrichment analysis. Top results with the false discovery rate (FDR) ≤0. 05 were considered noteworthy. The enriched pathways and processes were visualized in the volcano plot, in which the x and y axis indicated the enrichment ratio and the log of the FDR for all the functional categories in the database [50, 51]. Next, we conducted Gene Set Enrichment Analysis (GSEA) to uncover the signaling pathways and biological processes in which differentially expressed genes between high and low risk subgroups were enriched (http://software.broadinstitute.org/gsea/).

### Development of nomogram

Age, gender, stage, T, N, M, and risk score were used to construct a nomogram, using the survival and the rms package for R. Following that, calibration curves were plotted to evaluate the concordance between actual and predicted survival. Moreover, the concordance index (*C*-index), ranging from 0.5 to 1.0, was computed to assess the model performance for predicting prognosis was measured. The value of 0.5 and 1.0 represents a random chance and an excellent capacity for predicting survival with the model, respectively.

### Statistical analysis

All statistics were executed using the R software (Version 3.6.0; https://www.R-project.org). *x*^2^ test was used to check the association of risk scores with clinical characteristics. Kaplan-Meier curves were plotted and a log-rank test was used to check the significant difference in OS between groups. Univariate and multivariate Cox proportional hazard regression analysis was also performed to access the association between risk score and OS. The Receiver Operating Characteristic (ROC) analysis was used to examine the sensitivity and specificity of survival prediction using the gene signature risk score. An area under the ROC curve (AUC) served as an indicator of prognostic accuracy. A *P*-value of less than 0.05 was set as statistically significant for all the analyses.

## Supplementary Material

Supplementary Figures

Supplementary Table 1

## References

[1] Torre LA, Bray F, Siegel RL, Ferlay J, Lortet-Tieulent J, Jemal A. Global cancer statistics, 2012. CA Cancer J Clin. 2015; 65:87–108. 10.3322/caac.21262 25651787

[2] Chen W, Zheng R, Baade PD, Zhang S, Zeng H, Bray F, Jemal A, Yu XQ, He J. Cancer statistics in China, 2015. CA Cancer J Clin. 2016; 66:115–32. 10.3322/caac.21338 26808342

[3] Siegel RL, Miller KD, Jemal A. Cancer statistics, 2018. CA Cancer J Clin. 2018; 68:7–30. 10.3322/caac.21442 29313949

[4] Sharma K, Le N, Alotaibi M, Gewirtz DA. Cytotoxic autophagy in cancer therapy. Int J Mol Sci. 2014; 15:10034–51. 10.3390/ijms150610034 24905404 PMC4100138

[5] Marinković M, Šprung M, Buljubašić M, Novak I. Autophagy Modulation in Cancer: Current Knowledge on Action and Therapy. Oxid Med Cell Longev. 2018; 2018:8023821. 10.1155/2018/8023821 29643976 PMC5831833

[6] Levy JM, Towers CG, Thorburn A. Targeting autophagy in cancer. Nat Rev Cancer. 2017; 17:528–42. 10.1038/nrc.2017.53 28751651 PMC5975367

[7] Guo JY, White E. Autophagy, Metabolism, and Cancer. Cold Spring Harb Symp Quant Biol. 2016; 81:73–78. 10.1101/sqb.2016.81.030981 28209717 PMC5521269

[8] Duffy A, Le J, Sausville E, Emadi A. Autophagy modulation: a target for cancer treatment development. Cancer Chemother Pharmacol. 2015; 75:439–47. 10.1007/s00280-014-2637-z 25422156

[9] Guo JY, White E. Autophagy is required for mitochondrial function, lipid metabolism, growth, and fate of KRAS(G12D)-driven lung tumors. Autophagy. 2013; 9:1636–38. 10.4161/auto.26123 23959381 PMC5424446

[10] Strohecker AM, Guo JY, Karsli-Uzunbas G, Price SM, Chen GJ, Mathew R, McMahon M, White E. Autophagy sustains mitochondrial glutamine metabolism and growth of BrafV600E-driven lung tumors. Cancer Discov. 2013; 3:1272–85. 10.1158/2159-8290.CD-13-0397 23965987 PMC3823822

[11] Wang X, Li W, Zhang N, Zheng X, Jing Z. Opportunities and challenges of co-targeting epidermal growth factor receptor and autophagy signaling in non-small cell lung cancer. Oncol Lett. 2019; 18:499–506. 10.3892/ol.2019.10372 31289521 PMC6546992

[12] Sui X, Kong N, Zhu M, Wang X, Lou F, Han W, Pan H. Cotargeting EGFR and autophagy signaling: A novel therapeutic strategy for non-small-cell lung cancer. Mol Clin Oncol. 2014; 2:8–12. 10.3892/mco.2013.187 24649300 PMC3915646

[13] Henson E, Chen Y, Gibson S. EGFR Family Members’ Regulation of Autophagy Is at a Crossroads of Cell Survival and Death in Cancer. Cancers (Basel). 2017; 9:E27. 10.3390/cancers9040027 28338617 PMC5406702

[14] Han W, Pan H, Chen Y, Sun J, Wang Y, Li J, Ge W, Feng L, Lin X, Wang X, Wang X, Jin H. EGFR tyrosine kinase inhibitors activate autophagy as a cytoprotective response in human lung cancer cells. PLoS One. 2011; 6:e18691. 10.1371/journal.pone.0018691 21655094 PMC3107207

[15] Xie K, Liang C, Li Q, Yan C, Wang C, Gu Y, Zhu M, Du F, Wang H, Dai J, Liu X, Jin G, Shen H, et al. Role of ATG10 expression quantitative trait loci in non-small cell lung cancer survival. Int J Cancer. 2016; 139:1564–73. 10.1002/ijc.30205 27225307

[16] Aguirre-Gamboa R, Gomez-Rueda H, Martínez-Ledesma E, Martínez-Torteya A, Chacolla-Huaringa R, Rodriguez-Barrientos A, Tamez-Peña JG, Treviño V. SurvExpress: an online biomarker validation tool and database for cancer gene expression data using survival analysis. PLoS One. 2013; 8:e74250. 10.1371/journal.pone.0074250 24066126 PMC3774754

[17] Okayama H, Kohno T, Ishii Y, Shimada Y, Shiraishi K, Iwakawa R, Furuta K, Tsuta K, Shibata T, Yamamoto S, Watanabe S, Sakamoto H, Kumamoto K, et al. Identification of genes upregulated in ALK-positive and EGFR/KRAS/ALK-negative lung adenocarcinomas. Cancer Res. 2012; 72:100–11. 10.1158/0008-5472.CAN-11-1403 22080568

[18] Rousseaux S, Debernardi A, Jacquiau B, Vitte AL, Vesin A, Nagy-Mignotte H, Moro-Sibilot D, Brichon PY, Lantuejoul S, Hainaut P, Laffaire J, de Reyniès A, Beer DG, et al. Ectopic activation of germline and placental genes identifies aggressive metastasis-prone lung cancers. Sci Transl Med. 2013; 5:186ra66. 10.1126/scitranslmed.3005723 23698379 PMC4818008

[19] Bild AH, Yao G, Chang JT, Wang Q, Potti A, Chasse D, Joshi MB, Harpole D, Lancaster JM, Berchuck A, Olson JA Jr, Marks JR, Dressman HK, et al. Oncogenic pathway signatures in human cancers as a guide to targeted therapies. Nature. 2006; 439:353–57. 10.1038/nature04296 16273092

[20] Lee ES, Son DS, Kim SH, Lee J, Jo J, Han J, Kim H, Lee HJ, Choi HY, Jung Y, Park M, Lim YS, Kim K, et al. Prediction of recurrence-free survival in postoperative non-small cell lung cancer patients by using an integrated model of clinical information and gene expression. Clin Cancer Res. 2008; 14:7397–404. 10.1158/1078-0432.CCR-07-4937 19010856

[21] Won YW, Joo J, Yun T, Lee GK, Han JY, Kim HT, Lee JS, Kim MS, Lee JM, Lee HS, Zo JI, Kim S. A nomogram to predict brain metastasis as the first relapse in curatively resected non-small cell lung cancer patients. Lung Cancer. 2015; 88:201–07. 10.1016/j.lungcan.2015.02.006 25726044

[22] Liang W, Zhang L, Jiang G, Wang Q, Liu L, Liu D, Wang Z, Zhu Z, Deng Q, Xiong X, Shao W, Shi X, He J. Development and validation of a nomogram for predicting survival in patients with resected non-small-cell lung cancer. J Clin Oncol. 2015; 33:861–69. 10.1200/JCO.2014.56.6661 25624438

[23] Valentini V, van Stiphout RG, Lammering G, Gambacorta MA, Barba MC, Bebenek M, Bonnetain F, Bosset JF, Bujko K, Cionini L, Gerard JP, Rödel C, Sainato A, et al. Nomograms for predicting local recurrence, distant metastases, and overall survival for patients with locally advanced rectal cancer on the basis of European randomized clinical trials. J Clin Oncol. 2011; 29:3163–72. 10.1200/JCO.2010.33.1595 21747092

[24] Han DS, Suh YS, Kong SH, Lee HJ, Choi Y, Aikou S, Sano T, Park BJ, Kim WH, Yang HK. Nomogram predicting long-term survival after d2 gastrectomy for gastric cancer. J Clin Oncol. 2012; 30:3834–40. 10.1200/JCO.2012.41.8343 23008291

[25] Karakiewicz PI, Briganti A, Chun FK, Trinh QD, Perrotte P, Ficarra V, Cindolo L, De la Taille A, Tostain J, Mulders PF, Salomon L, Zigeuner R, Prayer-Galetti T, et al. Multi-institutional validation of a new renal cancer-specific survival nomogram. J Clin Oncol. 2007; 25:1316–22. 10.1200/JCO.2006.06.1218 17416852

[26] Überall I, Gachechiladze M, Joerger M, Anděl J, Smičková P, Kolek V, Grygárková I, Škarda J. Tumor autophagy is associated with survival outcomes in patients with resected non-small cell lung cancer. Lung Cancer. 2019; 129:85–91. 10.1016/j.lungcan.2019.01.001 30797498

[27] Chen HY, Yu SL, Chen CH, Chang GC, Chen CY, Yuan A, Cheng CL, Wang CH, Terng HJ, Kao SF, Chan WK, Li HN, Liu CC, et al. A five-gene signature and clinical outcome in non-small-cell lung cancer. N Engl J Med. 2007; 356:11–20. 10.1056/NEJMoa060096 17202451

[28] Wang Y, Zhang Q, Gao Z, Xin S, Zhao Y, Zhang K, Shi R, Bao X. A novel 4-gene signature for overall survival prediction in lung adenocarcinoma patients with lymph node metastasis. Cancer Cell Int. 2019; 19:100. 10.1186/s12935-019-0822-1 31015800 PMC6469135

[29] Xiong Y, Wang R, Peng L, You W, Wei J, Zhang S, Wu X, Guo J, Xu J, Lv Z, Fu Z. An integrated lncRNA, microRNA and mRNA signature to improve prognosis prediction of colorectal cancer. Oncotarget. 2017; 8:85463–78. 10.18632/oncotarget.20013 29156733 PMC5689623

[30] Mo S, Dai W, Xiang W, Li Y, Feng Y, Zhang L, Li Q, Cai G. Prognostic and predictive value of an autophagy-related signature for early relapse in stages I-III colon cancer. Carcinogenesis. 2019; 40:861–70. 10.1093/carcin/bgz031 30933267

[31] Chai RC, Wu F, Wang QX, Zhang S, Zhang KN, Liu YQ, Zhao Z, Jiang T, Wang YZ, Kang CS. m^6^A RNA methylation regulators contribute to malignant progression and have clinical prognostic impact in gliomas. Aging (Albany NY). 2019; 11:1204–25. 10.18632/aging.101829 30810537 PMC6402513

[32] Zhou Z, Mo S, Dai W, Ying Z, Zhang L, Xiang W, Han L, Wang Z, Li Q, Wang R, Cai G. Development and Validation of an Autophagy Score Signature for the Prediction of Post-operative Survival in Colorectal Cancer. Front Oncol. 2019; 9:878. 10.3389/fonc.2019.00878 31552190 PMC6746211

[33] Lin P, He RQ, Dang YW, Wen DY, Ma J, He Y, Chen G, Yang H. An autophagy-related gene expression signature for survival prediction in multiple cohorts of hepatocellular carcinoma patients. Oncotarget. 2018; 9:17368–95. 10.18632/oncotarget.24089 29707114 PMC5915122

[34] An Y, Bi F, You Y, Liu X, Yang Q. Development of a Novel Autophagy-related Prognostic Signature for Serous Ovarian Cancer. J Cancer. 2018; 9:4058–71. 10.7150/jca.25587 30410611 PMC6218776

[35] Kwon Y, Kim M, Jung HS, Kim Y, Jeoung D. Targeting Autophagy for Overcoming Resistance to Anti-EGFR Treatments. Cancers (Basel). 2019; 11:E1374. 10.3390/cancers11091374 31527477 PMC6769649

[36] Liu G, Pei F, Yang F, Li L, Amin AD, Liu S, Buchan JR, Cho WC. Role of Autophagy and Apoptosis in Non-Small-Cell Lung Cancer. Int J Mol Sci. 2017; 18:E367. 10.3390/ijms18020367 28208579 PMC5343902

[37] Mariño G, Niso-Santano M, Baehrecke EH, Kroemer G. Self-consumption: the interplay of autophagy and apoptosis. Nat Rev Mol Cell Biol. 2014; 15:81–94. 10.1038/nrm3735 24401948 PMC3970201

[38] Delgado ME, Dyck L, Laussmann MA, Rehm M. Modulation of apoptosis sensitivity through the interplay with autophagic and proteasomal degradation pathways. Cell Death Dis. 2014; 5:e1011. 10.1038/cddis.2013.520 24457955 PMC4040655

[39] Zhang M, Su L, Xiao Z, Liu X, Liu X. Methyl jasmonate induces apoptosis and pro-apoptotic autophagy via the ROS pathway in human non-small cell lung cancer. Am J Cancer Res. 2016; 6:187–99. 27186395 PMC4859652

[40] Wei Y, Pattingre S, Sinha S, Bassik M, Levine B. JNK1-mediated phosphorylation of Bcl-2 regulates starvation-induced autophagy. Mol Cell. 2008; 30:678–88. 10.1016/j.molcel.2008.06.001 18570871 PMC2478643

[41] Wu H, Che X, Zheng Q, Wu A, Pan K, Shao A, Wu Q, Zhang J, Hong Y. Caspases: a molecular switch node in the crosstalk between autophagy and apoptosis. Int J Biol Sci. 2014; 10:1072–83. 10.7150/ijbs.9719 25285039 PMC4183927

[42] Oral O, Oz-Arslan D, Itah Z, Naghavi A, Deveci R, Karacali S, Gozuacik D. Cleavage of Atg3 protein by caspase-8 regulates autophagy during receptor-activated cell death. Apoptosis. 2012; 17:810–20. 10.1007/s10495-012-0735-0 22644571

[43] Li T, Su L, Zhong N, Hao X, Zhong D, Singhal S, Liu X. Salinomycin induces cell death with autophagy through activation of endoplasmic reticulum stress in human cancer cells. Autophagy. 2013; 9:1057–68. 10.4161/auto.24632 23670030 PMC3722315

[44] Nam T, Han JH, Devkota S, Lee HW. Emerging Paradigm of Crosstalk between Autophagy and the Ubiquitin-Proteasome System. Mol Cells. 2017; 40:897–905. 10.14348/molcells.2017.0226 29237114 PMC5750708

[45] Tian X, Zhu X, Yan T, Yu C, Shen C, Hu Y, Hong J, Chen H, Fang JY. Recurrence-associated gene signature optimizes recurrence-free survival prediction of colorectal cancer. Mol Oncol. 2017; 11:1544–60. 10.1002/1878-0261.12117 28796930 PMC5664005

[46] Zhang JX, Song W, Chen ZH, Wei JH, Liao YJ, Lei J, Hu M, Chen GZ, Liao B, Lu J, Zhao HW, Chen W, He YL, et al. Prognostic and predictive value of a microRNA signature in stage II colon cancer: a microRNA expression analysis. Lancet Oncol. 2013; 14:1295–306. 10.1016/S1470-2045(13)70491-1 24239208

[47] Friedman J, Hastie T, Tibshirani R. Regularization Paths for Generalized Linear Models via Coordinate Descent. J Stat Softw. 2010; 33:1–22. 10.18637/jss.v033.i01 20808728 PMC2929880

[48] Sauerbrei W, Royston P, Binder H. Selection of important variables and determination of functional form for continuous predictors in multivariable model building. Stat Med. 2007; 26:5512–28. 10.1002/sim.3148 18058845

[49] Kidd AC, McGettrick M, Tsim S, Halligan DL, Bylesjo M, Blyth KG. Survival prediction in mesothelioma using a scalable Lasso regression model: instructions for use and initial performance using clinical predictors. BMJ Open Respir Res. 2018; 5:e000240. 10.1136/bmjresp-2017-000240 29468073 PMC5812388

[50] Liao Y, Wang J, Jaehnig EJ, Shi Z, Zhang B. WebGestalt 2019: gene set analysis toolkit with revamped UIs and APIs. Nucleic Acids Res. 2019; 47:W199–205. 10.1093/nar/gkz401 31114916 PMC6602449

[51] Zhang B, Kirov S, Snoddy J. WebGestalt: an integrated system for exploring gene sets in various biological contexts. Nucleic Acids Res. 2005; 33:W741–8. 10.1093/nar/gki475 15980575 PMC1160236

