# Supplementary material for: Prognostic implications of autophagy-associated gene signatures in non-small cell lung cancer
Source: Aging (Albany NY). 2019 Dec 7;11(23):11440–62. doi: 10.18632/aging.102544 (PMC6932887; doi:10.18632/aging.102544)
Supplement: Supplementary Figures [file aging-11-102544-s002..pdf]

SUPPLEMENTARY FIGURES

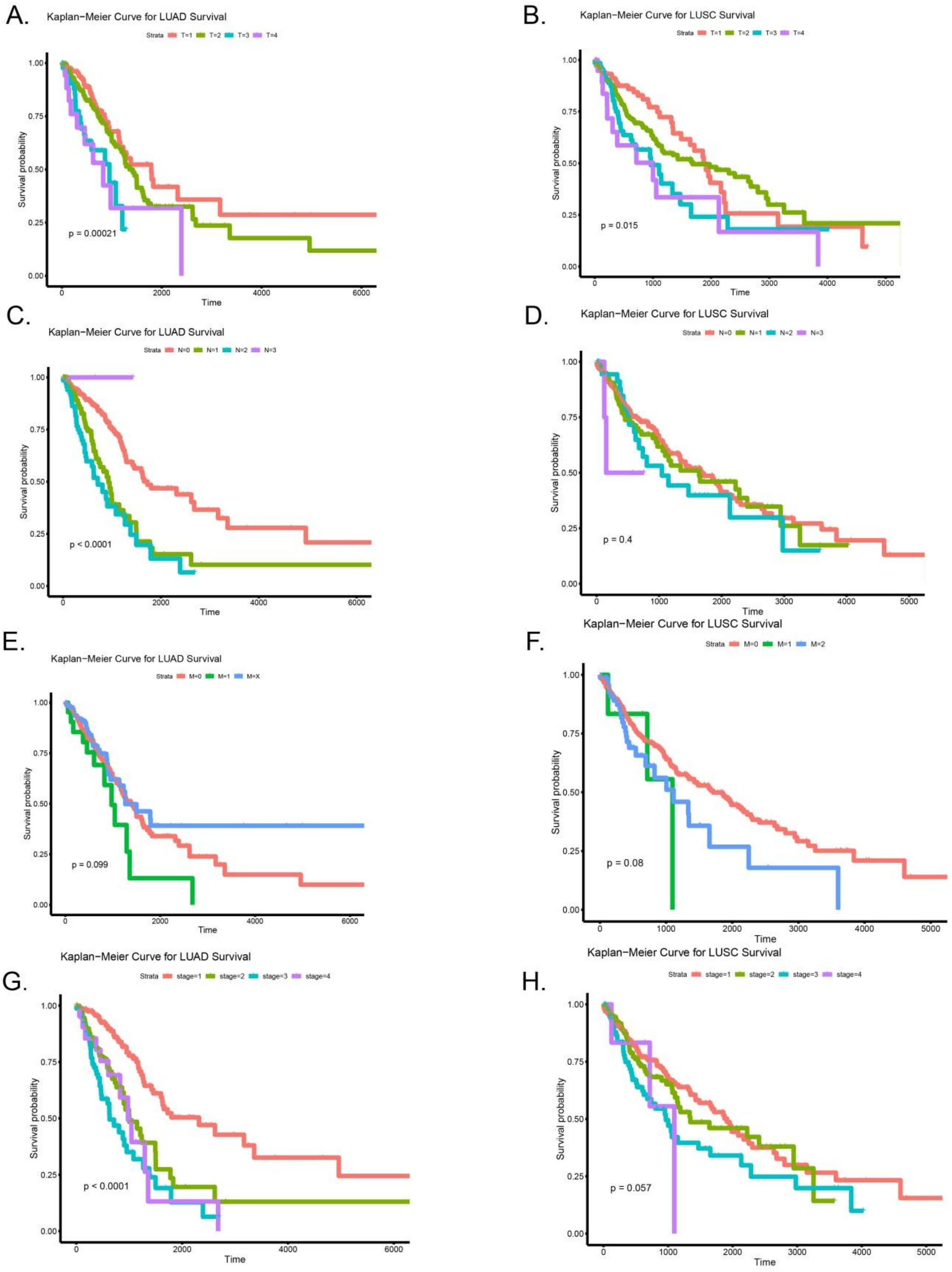

Supplementary Figure 1. Kaplan-Meier survival curves regarding the clinical features in TCGA lung cancer cohorts. Survival curves for LUAD (**A**, **C**, **E**, and **G**) and LUSC (**B**, **D**, **F**, and **H**) were exhibited on the left and right panels, respectively.

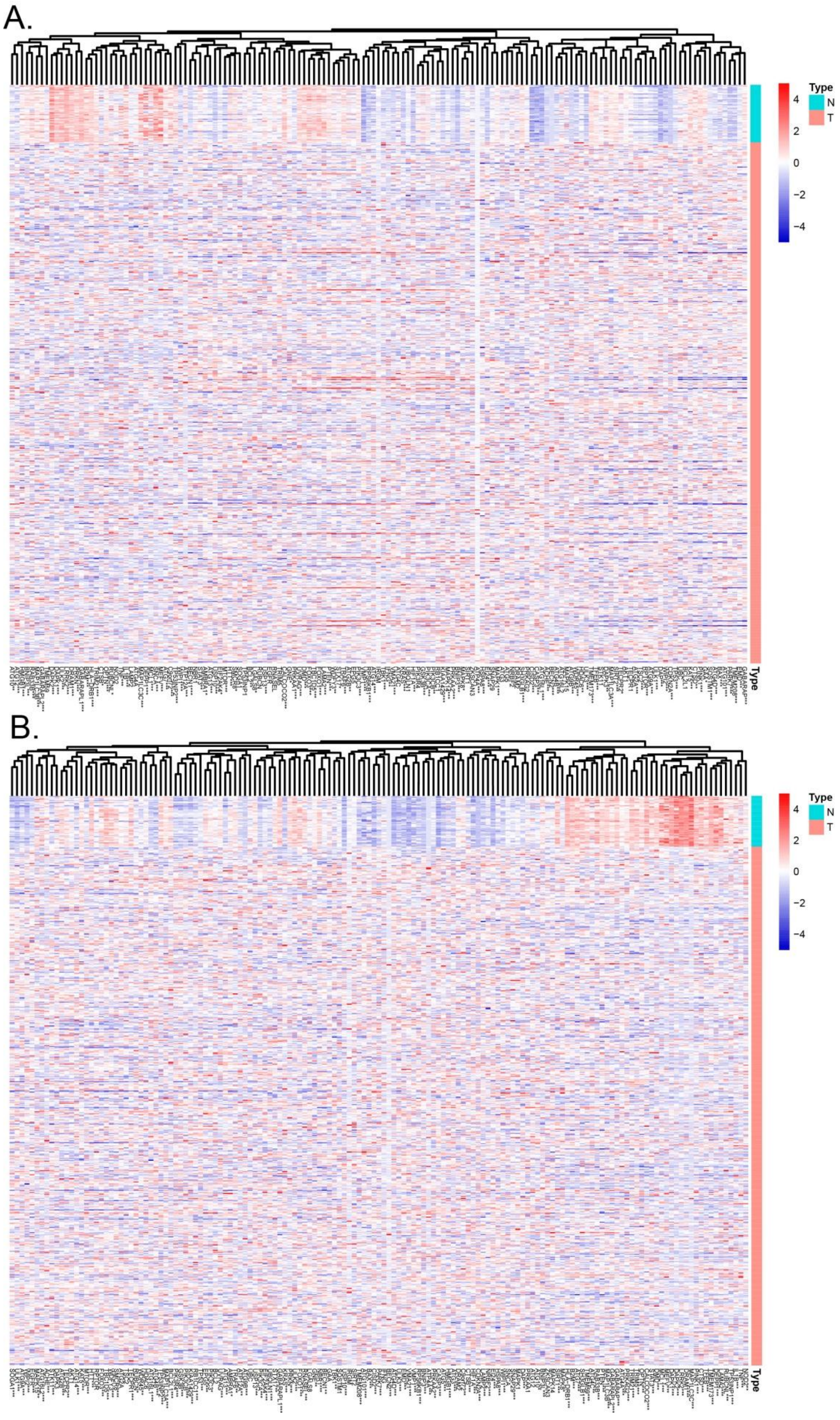

Supplementary Figure 2. mRNA expression profiles of 148 autophagy-associated genes in TCGA lung cancer cohorts. Gene expression profiles for LUAD (**A**) and LUSC (**B**) were exhibited in the up and low panels, respectively.

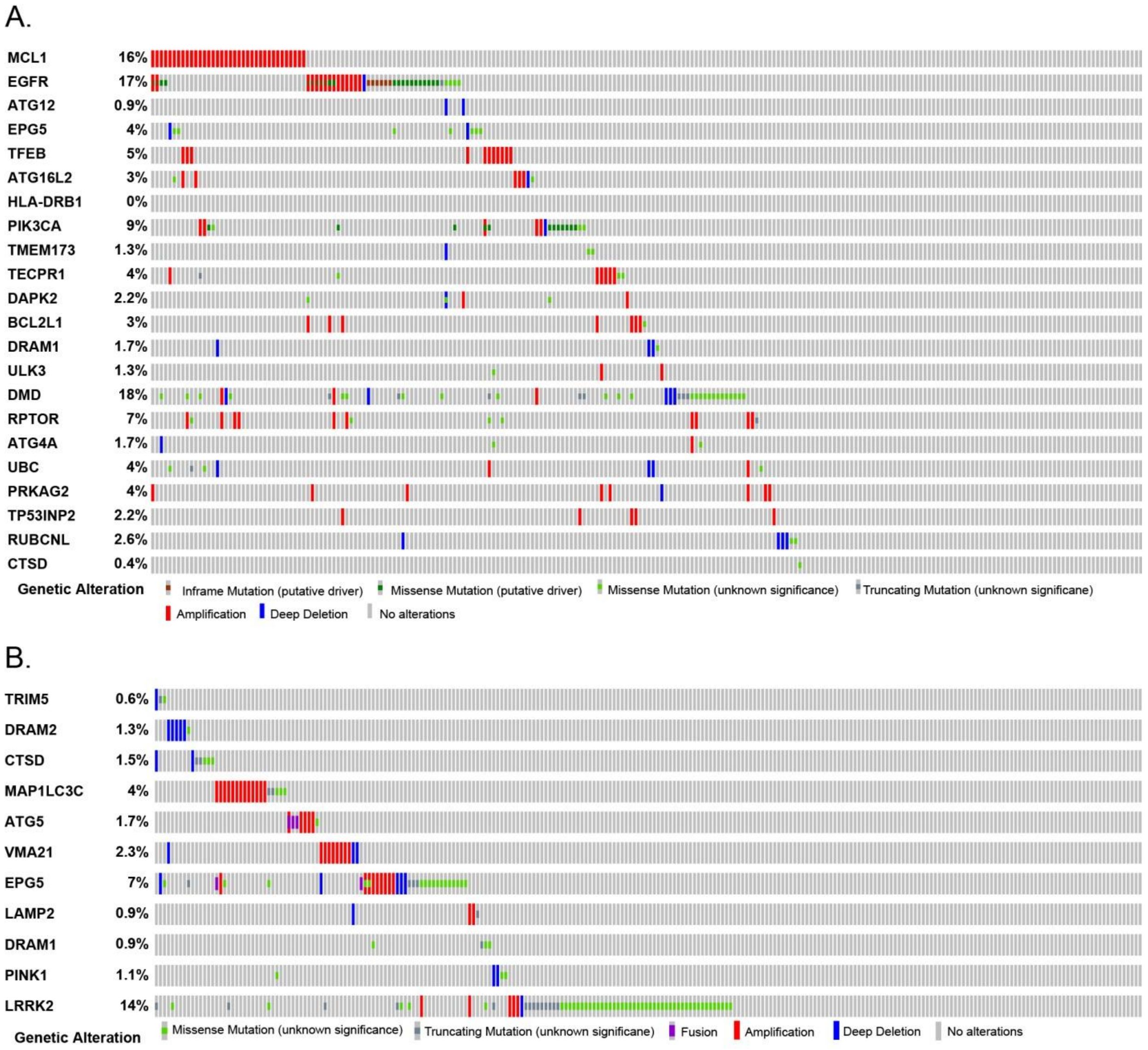

Supplementary Figure 3. Genetic alteration of genes included in the gene signatures (TCGA, PanCancer Atlas). (**A**) Genetic alteration of the 22 genes in the TCGA-LUAD cohort. (**B**) Genetic alteration of the 11 genes in the TCGA-LUSC cohort.
