# Supplementary material for: Prognostic implications of autophagy-associated gene signatures in non-small cell lung cancer
Source: Aging (Albany NY). 2019 Dec 7;11(23):11440–62. doi: 10.18632/aging.102544 (PMC6932887; doi:10.18632/aging.102544)
Supplement: Supplementary Table 1 [file aging-11-102544-s001..pdf]

SUPPLEMENTARY TABLE

Supplementary Table 1. Characteristics of TCGA lung cancer cohorts.

		LUAD	LUSC
**Cases**		490	488
**Age**			
	≤65	237	189
	>65	253	299
**Gender**			
	Female	266	127
	Male	224	361
**Stage**			
	Stage I	261	238
	Stage II	117	156
	Stage III	79	83
	Stage IV	25	7
**T(Tumor)**			
	T1	166	110
	T2	258	285
	T3	45	70
	T4	18	23
**N(Lymph Node)**			
	N0	317	312
	N1	92	126
	N2	68	40
	N3	2	5
**M(Metastasis)**			
	M0	322	400
	M1	24	7
	MX	140	77
